# Reversing Stimulus Timing in Visual Conditioning Leads to Memories with Opposite Valence in *Drosophila*

**DOI:** 10.1371/journal.pone.0139797

**Published:** 2015-10-02

**Authors:** Katrin Vogt, Ayse Yarali, Hiromu Tanimoto

**Affiliations:** 1 Max Planck Institute of Neurobiology, 82152, Martinsried, Germany; 2 Leibniz Institute for Neurobiology (LIN), Research Group Molecular Systems Biology of Learning, 39118, Magdeburg, Germany; 3 Center for Behavioral Brain Sciences, 39118, Magdeburg, Germany; 4 Tohoku University Graduate School of Life Sciences, 980–8577, Sendai, Japan; Tokai University, JAPAN

## Abstract

Animals need to associate different environmental stimuli with each other regardless of whether they temporally overlap or not. *Drosophila melanogaster* displays olfactory trace conditioning, where an odor is followed by electric shock reinforcement after a temporal gap, leading to conditioned odor avoidance. Reversing the stimulus timing in olfactory conditioning results in the reversal of memory valence such that an odor that follows shock is later on approached (i.e. relief conditioning). Here, we explored the effects of stimulus timing on memory in another sensory modality, using a visual conditioning paradigm. We found that flies form visual memories of opposite valence depending on stimulus timing and can associate a visual stimulus with reinforcement despite being presented with a temporal gap. These results suggest that associative memories with non-overlapping stimuli and the effect of stimulus timing on memory valence are shared across sensory modalities.

## Introduction

Animals associate sensory stimuli in their environment with concurrent reinforcement in order to generate anticipatory behavior. Interestingly, perfect stimulus-reinforcement overlap does not lead to the strongest associative memories; instead the greatest memory effects are found when a stimulus precedes reinforcement with a partial temporal overlap, highlighting the predictive nature of associative learning [1]. Interestingly, temporal overlap between stimulus and reinforcement is dispensable for associative learning. For example, during conditioned taste aversion, an animal associates a consumed food item with the “feeling of sickness” occurring hours after ingestion; and this food item is avoided during subsequent encounters [2,3]. In addition to stimuli that precede an aversive reinforcement, the stimuli that follow the aversive reinforcement are relevant, since gaining preference towards these stimuli may help to keep the exposure to a threat at a minimum (for a discussion within the framework of the threat-imminence model, see [4]).

*Drosophila* is an excellent model for studying the role of stimulus timing in associative memory, given its experimental accessibility at molecular, cellular and behavioral levels. Indeed adult and larval *Drosophila* can form associations between an odor and subsequent electric shock, even when these two are separated by a temporal gap (i.e., trace conditioning), such that they later avoid the trained odor [5–8]. When stimulus timing is reversed during training, such that the odor follows the electric shock, flies form an oppositely valenced memory and subsequently approach this odor [6,9,10]. It has been proposed that during this kind of training, the odor becomes a predictor for the “feeling of relief” [11,12] (for discussion of alternative safety-based explanations, see [4,13,14]; for the corresponding effect of stimulus timing on appetitive olfactory conditioning in honey bee, see [15]). Thus, one kind of reinforcement, i.e., electric shock, can establish two opposite conditioned behaviors towards an odor, depending on stimulus timing.

In this study, we systematically characterized the effect of stimulus timing in visual conditioning using a classical conditioning paradigm with chromatic visual cues and electric shock reinforcement [16]. Visual stimuli lend themselves to better temporal control than olfactory stimuli [7]. In human and rodent subjects, training with non-overlapping visual stimuli and electric shock reinforcement leads to opponent memories depending on stimulus timing (i.e. trace *vs*. relief conditioning) [17,18]. Can insects perform such visual trace and relief conditioning tasks? Honeybees clearly maintain a trace of visual information in free flight [19–23]; whereas *Drosophila* preserves a trace of visual stimuli at least for orientation purposes [24]. However, it has not been tested whether insects can use traces of visual stimuli for formation of associative memories and whether reversing the stimulus timing influences the associative valence in insects in the visual modality.

In order to tackle these questions in *Drosophila*, we first optimized the training parameters in our recently developed visual conditioning paradigm [16]. We then systematically varied the relative timing of visual stimulus and reinforcement, to probe for visual trace and relief memories.

## Materials & Methods

### Flies

*Drosophila melanogaster* were reared in mass culture at 25°C, at 60% relative humidity, under a 12–12-hour light-dark cycle on a standard cornmeal-based diet. The Canton-Special (CS) wild type strain was used for all experiments. Before experiments, 1–3 day old flies were collected in fresh food vials and kept overnight at 25°C and 60% relative humidity. Each behavioral experiment employed 30–40 flies of mixed gender and was performed in dim red light.

### Visual conditioning setup

Computer-controlled LED arrays were used to generate visual stimuli (green/blue light: 452 nm and 520 nm (Seoul Z-Power RGB LED) or 456 nm and 520 nm (H-HP803NB, and H-HP803PG, 3W Hexagon Power LEDs, Roithner Lasertechnik)) from beneath the flies [16,25]. LEDs were fitted in a substructure (165 mm below the arena), which allowed homogeneous illumination of a piece of filter paper used as a screen. The light intensities were controlled by current and calibrated using a luminance meter BM-9 (Topcon Technohouse Corporation) or a PR-655 SpectraScan® Spectroradiometer: 14.1 Cd/m^2^ s (blue) and 70.7 Cd/m^2^ s (green) [16,25]. To deliver electric shock punishment, a custom made arena with a transparent shock grid was used [16]. The shock grid was composed of a glass plate (9x9 cm) with a laser-structured transparent ITO grid (1.6 mm width with 0.1 mm of gaps). A plastic ring (wall) and a glass lid for the arena were coated with diluted Fluon (10%; Fluon GP1, Whitford Plastics Ltd., UK) to prevent flies from walking on the lid and wall. Consequently, flies were forced to stay on the shock grid at the bottom of the arena. To analyze data from behavioral experiments, the distribution of the flies was recorded during the test phase from above with a CMOS camera (Firefly MV, Point Grey, Richmond, Canada) controlled by custom made software [26]. Four setups were run in parallel.

### Behavioral protocol

**Fig 1:**
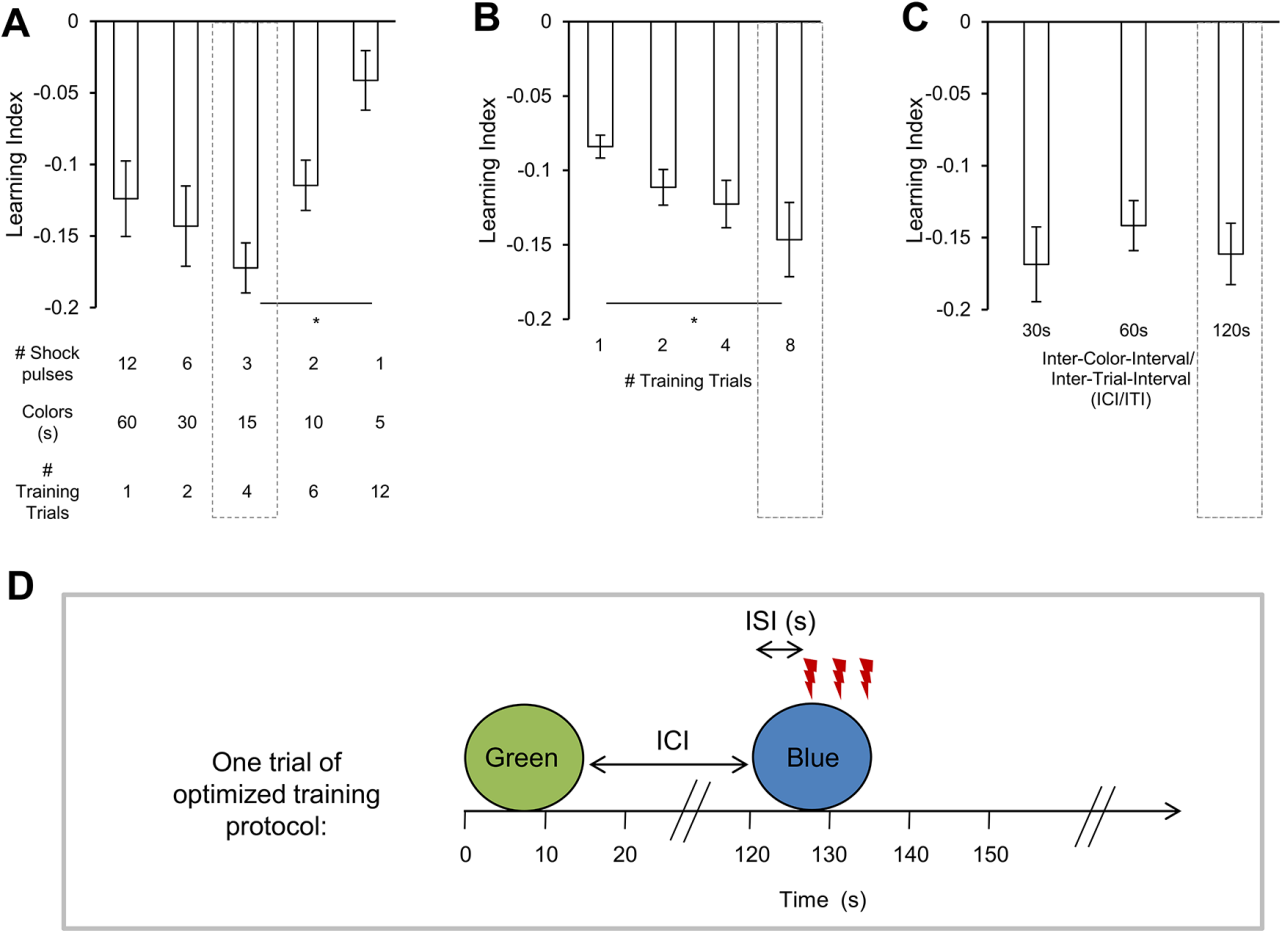
Optimizing the training protocol. In (A-C), the paired color presentation preceded the electric shock application by 4 s. (inter-stimulus-interval (ISI) = -4s). (A) To find the best parameters for visual conditioning we varied the number of training trials, however each experimental group received the same total number of electric shock pulses (12) and the same total duration of color presentation (60 s per color). Visual learning scores depended on such variation in protocol (Kruskal-Wallis test, H = 15.02, d.f. = 4, p < 0.005). Significant difference in scores was found when comparing 4 trial- *vs*. 12 trial-training (*post-hoc* pairwise comparison, p < 0.05). Flies showed significant scores after 1, 2, 4 and 6 trials (one sample *t*-tests, T > 4.7, p < 0.001). Applying 12 trials with 5 s of color presentation and 1 electric shock pulse did not reveal significant conditioned avoidance (one sample *t*-test, T = 1.99, p > 0.1), *n* = 15–20. (B) Using the optimal conditions from (A) (dashed box), application of one to eight training trials led to significant conditioned avoidance (one-sample Wilcoxon signed rank tests, p < 0.001). Significant difference in scores was found between conditioning with one trial *vs*. 8 trials (Kruskal Wallis test, *post-hoc* pairwise comparison p < 0.05), *n* = 16 (C) Using the optimal conditions from (A) and (B) (dashed boxes), we varied the inter-color-interval (ICI) and inter-trial-interval (ITI) between 30 s and 120 s. Learning scores did not depend on the duration of the ICI or ITI (one-way ANOVA, F = 0.407, p > 0.6). All groups showed significant scores (one sample *t*-tests, T > 6.2, p < 0.001), *n* = 20. In (A-C), bars and error bars represent means and SEMs, respectively. (D) The resulting optimized training protocol with 8 training trials; each with 15 s long presentations of color stimuli, 3 electric shock pulses and an ICI and ITI of 120 s. Only one trial is sketched.

**Fig 2:**
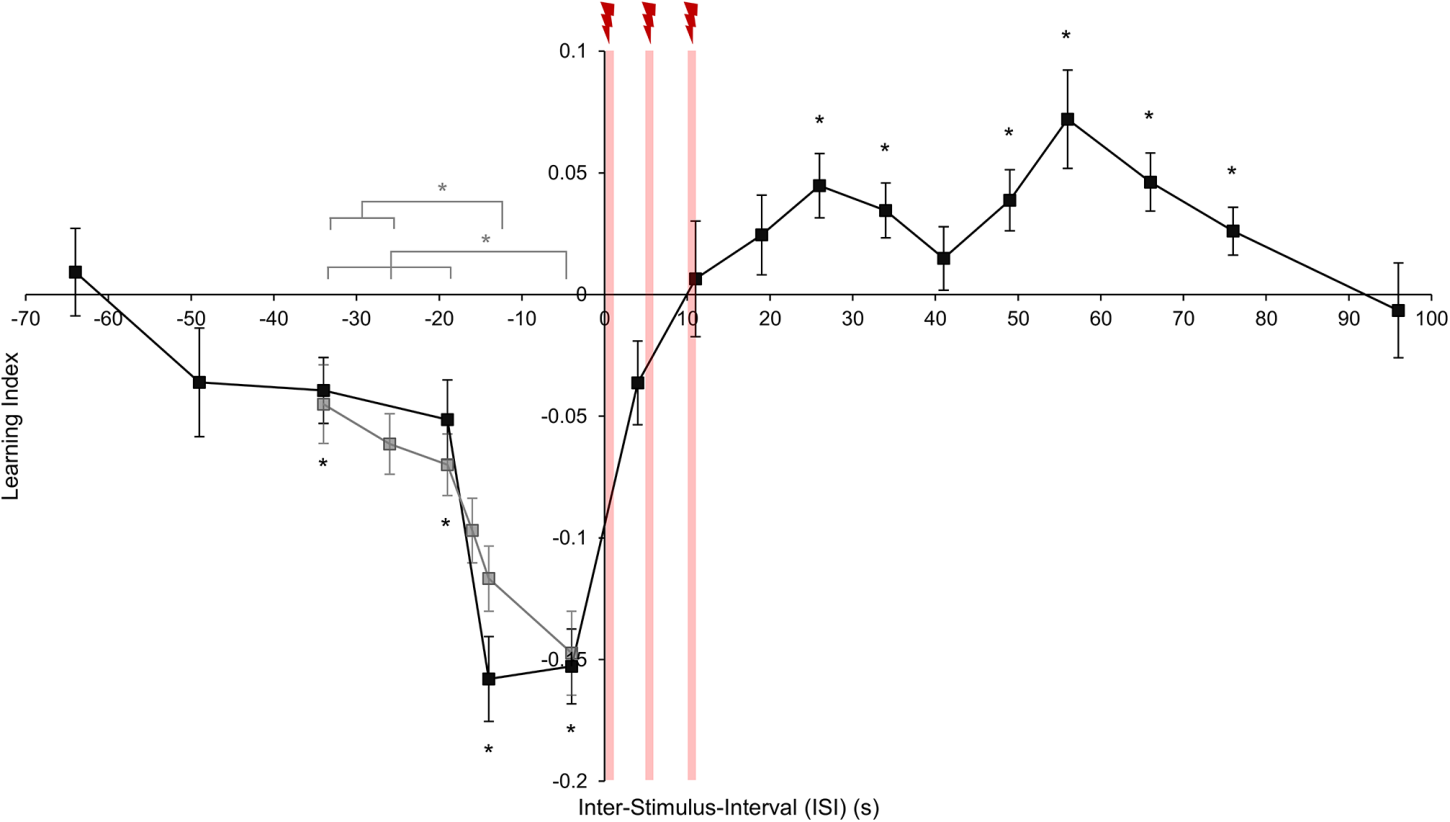
Effect of stimulus timing on visual memory. Conditioned behavior as a function of inter-stimulus interval (ISI). Red stripes indicate electric shock pulses. Data points indicate onset of the paired color presentation with 15 s duration (x-axis) and mean learning index (y-axis). Error bars represent the SEMs. Black data points: Learning scores depended on the ISI (one-way ANOVA, F = 13.86, p < 0.001). Flies showed significant conditioned avoidance with overlapping paired color presentation and shock pulses (one sample *t*-tests, T > 8.3, p < 0.001, ISI = -14 s, -4 s) and when the paired color preceded shock by a short temporal gap (one sample *t*-tests, T > 2.9, p < 0.01; trace conditioning, ISI = -34 s, -19 s). Flies showed significant conditioned approach when the paired color followed shock with a gap (one sample *t*-tests, T > 2.648, p < 0.05; relief conditioning, ISI = +26 s, +34 s, +49 s, +56 s, +66 s, +76 s; for ISI = +19 s and +41 s; T < 1.493; p > 0.05). When the paired color followed shock with overlap, scores did not differ from zero (one sample *t*-tests, T < 2.2, p > 0.05; ISI = +4 s, +11 s). Also when the two stimuli were too far apart in time (ISI = -64 s, -49 s, +96 s) flies showed no conditioned behavior (one sample *t*-tests, T < 1.7, p > 0.05), *n* = 12–44. Grey data points: We re-examined trace conditioning using additional negative ISIs (ISI = -34 s to -4 s). Flies showed significant conditioned avoidance with all tested ISI values (one sample *t*-tests, T > 3.0, p < 0.05). Learning scores depended on ISI value (one-way ANOVA, F = 7.355, p < 0.0001), such that the visual memory steadily decreased with increasing temporal gap (*post-hoc* pairwise comparisons p < 0.05 for -34 s *vs*. -14 s, -34 s *vs*. -4 s, -26 s *vs*. -14 s, -26 s *vs*. -4 s, -19 s *vs*. -4 s), *n* = 16–24.

We applied differential conditioning followed by a binary choice test [16,25,26]. Approximately 40 flies were introduced into the arena using an aspirator and subjected to a single conditioning experiment. During training the whole arena was illuminated alternately with green and blue light, one of which was paired with electric shock. Conditioning has been shown to be reproducible with a protocol using 60 s of green and blue color presentation and 12 pulses of electric shock per trial [16]. We optimized this conditioning protocol to facilitate variation in stimulus timing. We varied exposure to either color between 5 s and 60 s per trial (Fig 1A). As reinforcement, 1 to 12 1 s-long pulses of electric shock (AC 60 V) were applied every 5 s (Fig 1A). The extent of training varied between 1 to 12 trials (Fig 1A and 1B). Two consecutive presentations of paired color and unpaired color were separated by a dark inter-color-interval (ICI) and an inter-trial-interval (ITI) of 12–120 s (Fig 1A–1C). For the experiments where we used variable stimulus timing we employed the following parameter values: 15 s of green and blue color with 3 electric shock pulses per trial, eight training trials and an ICI as well as ITI of 120 s (Figs 1D, 2 and S1 Fig (here duration of color presentation varies from 5 to 25 s)). In protocol optimization experiments, the onset of the visual stimulus preceded the onset of electric shock punishment by 4 s (inter-stimulus-interval (ISI) = -4 s, Fig 1A–1C). For conditioning with variable stimulus timing, different groups of flies were trained with different ISIs, negative numbers indicating that the onset of the paired color preceded the onset of the electric shock, positive numbers indicating that the onset of the paired color followed the onset of the electric shock (Fig 2 and S1 Fig).

In all experiments, the test started 60 s after the end of training. Unlike in the training phase, two diagonal quadrants of the arena were illuminated with green whereas the other two quadrants were illuminated with blue, to allow flies to choose between the two colors. The distribution of the flies was recorded for 90 s at 1 frame per second [26]. No electric shock was presented in the test; however a 1 s shock pulse (AC 90V) was applied 5 s before the beginning of the test to arouse the flies [16]. Two groups undergoing reciprocal contiguity for colors and electric shock (Green+/Blue− and Blue+/Green−) were trained in the same setup consecutively. A preference index (PI) was calculated for each group: PI = (# of flies on green quadrants–# of flies on blue quadrants) / Total # of flies. Thus, negative PI values indicate a preference for blue against green; whereas positive values indicate *vice versa*. The difference between the reciprocal groups in PI was then used to calculate a learning index (LI) for each video frame [26]: LI = (PI_Green+/Blue−_−PI_Blue+/Green−_) / 2. Thus, negative LIs indicate conditioned avoidance of the paired color; whereas positive values indicate conditioned approach.

### Statistics

Statistical analyses were performed with the Prism5 software (GraphPad). Groups that did not violate the assumption of normal distribution (Shapiro-Wilk test) and homogeneity of variance (Bartlett’s test) were analyzed with parametric statistics: one-sample *t*-test or one-way analysis of variance (ANOVA) followed by planned pairwise multiple comparisons (Benjamini Hochberg). Datasets that did not fulfill the assumptions above were analyzed with non-parametric tests, such as one-sample Wilcoxon signed rank test or Kruskal–Wallis test followed by Dunn’s multiple pairwise comparison. The significance level of statistical tests was set to 0.05.

## Results

### Optimizing the training protocol

To find the best parameters for training, we first varied the duration of color presentation and electric shock reinforcement (Fig 1A). Various experimental groups were exposed to the same total duration of color presentation and the same number of shock pulses, but distributed over different numbers of trials. For every group the paired color preceded the shock by 4 s (inter-stimulus-interval (ISI) = -4 s). We found the lowest conditioned avoidance when flies were trained with 5 s-long color presentation, a single shock pulse per trial and 12 training trials. Increasing the number of shock pulses to three was not sufficient to improve scores with color presentation of 5 s (compare Fig 1A with S1 Fig), suggesting that the exposure to visual stimuli was too short. Maximum conditioned avoidance was found when color duration of 15 s was paired with three shock pulses, repeated over four trials. Prolongation of the color presentation to 25 s under these conditions did not improve learning scores (S1 Fig). When the optimal protocol of 15 s of color presentation and three pulses of shock was used, flies showed significant learning scores even with a single training trial (Fig 1B). However comparisons across one, two, four and eight training trials showed that visual conditioning did improve with training repetition (Fig 1B). Thus, in all further experiments eight training trials were applied. In order to exclude any associations between the electric shock and the unpaired color, we considered increasing the interval between the two consecutive colors (inter-color-interval (ICI)) and between training trials (inter-trial-interval (ITI)). We tested different ICIs/ ITIs (30 s, 60 s, 120 s; Fig 1C), but found no significant difference across groups. Thus, the protocol in further experiments consisted of eight training trials with 15 s of blue and green stimulation, three electric shock pulses and an ICI and ITI of 120s (Fig 1D).

### Effect of stimulus timing on visual memory

Using the conditions specified above, we trained independent groups of flies with ISIs varying from -64 s (the onset of the paired color preceded the onset of shock by 64 s) to +96 s (the onset of the paired color followed the onset of the shock by 96 s). No significant scores were found with very long ISIs in either direction (ISI = -64 s, -49 s and +96 s, Fig 2, black data points). When the paired color shortly preceded the shock flies acquired significant conditioned avoidance, independent of the duration of overlap between the two stimuli (ISI = -14 s and -4 s, Fig 2, black data points; see also S1 Fig). Critically, conditioned avoidance was found even when the paired color preceded the shock without overlap (ISI = -34 s and -19 s, Fig 2, black data points), but to a lesser extent. Thus, flies could indeed associate the trace of a visual cue with shock. To better resolve the temporal requirements for visual trace conditioning, we added further experimental groups with negative ISIs (Fig 2, grey data points), revealing that trace memory steadily weakened with increasing temporal gap between the offset of the paired color and the onset of the shock.

Interestingly, when the paired color followed the shock with overlap, flies showed weak or no conditioned avoidance (ISI = +4 s and +11 s, Fig 2, black data points). Critically however, when the paired color followed the shock with a temporal gap, flies approached the paired color in the test, suggesting relief conditioning (ISI = +19 s to +76 s, Fig 2, black data points). Thus, reversing stimulus timing indeed led to valence reversal in visual memory.

## Discussion

By using a classical conditioning setup with visual chromatic cues and electric shock reinforcement we showed that fruit flies form memories of opposite valence depending on the relative timing of stimuli during training, as is the case for the olfactory modality [6,9]. Furthermore, flies can associate a visual stimulus with a delayed and temporally non-overlapping aversive reinforcement, similar to effects reported in olfactory conditioning [6–8]. The shapes of the ISI-learning curves in both modalities are similar: Conditioning with negative ISIs generates a sharper response peak with stronger scores than conditioning with positive ISIs (compare Fig 2 to [6,27]). These parametric similarities across modalities may point to a common underlying molecular mechanism.

### Trace conditioning: visual *vs*. olfactory modalities

A caveat of the olfactory conditioning paradigm is that residues of the odor may remain in the setup, confounding trace conditioning experiments [7]. In experiments presented here, this concern does not apply. Therefore, the time gap between the paired color presentation and electric shock should be bridged by cellular and/ or molecular mechanisms [28]. In visual conditioning, our analyses revealed that: (i) a minimum duration for visual stimulus presentation is necessary to establish significant conditioned avoidance (Fig 1 and S1 Fig); (ii) temporal overlap between the visual stimulus and the reinforcement improves learning scores (Fig 2) and; (iii) when the onset of the visual stimulus precedes the onset of the reinforcement the strongest conditioned avoidance is established (Fig 2). These features are similar to those found in other associative learning paradigms [1,29]. Furthermore, in visual trace conditioning, we found the duration of the temporal gap between the visual stimulus and the reinforcement to be a strong determinant of conditioned avoidance: Prolonged duration of the time gap steadily decreased memory performance (Fig 2, grey data points), in agreement with key features of olfactory trace conditioning [7] and possibly pointing to a common underlying mechanism. This is not a trivial finding as the change in the saliency of the trace of a stimulus over time could instead have depended on the sensory modality in question [30]. In general, sustaining any kind of sensory trace is likely costly since it may require sustained neural activity [31]. The finding that fruit flies maintain a “costly” trace of stimuli from different sensory modalities suggests that such ability is highly adaptive.

The neural mechanisms underlying visual and olfactory conditioning are surprisingly similar in *Drosophila*. The same set of dopaminergic neurons is sufficient and necessary for signaling electric shock reinforcement to a shared set of mushroom body neurons in both cases [16,32–34]. Yet, it is still not known whether the same individual mushroom body intrinsic neurons serve both modalities or if there are dedicated subsets for each modality. Moreover, mushroom body neurons were suggested to maintain odor traces in olfactory conditioning [8,35]. Thus, it would be interesting to test whether this is also the case in visual conditioning.

### Relief conditioning: visual *vs*. olfactory modalities

As in olfactory conditioning [6,9,10], we found, for the first time, that in visual conditioning, flies approached the paired color when it followed the offset of shock reinforcement during training. In olfactory conditioning, a high number of training trials is required to induce such relief conditioning (> 4 trials [6,9,27]); applying eight training trials in our case resulted in good performance when flies were trained with colors (Figs 1B and 2). We applied few electric shock pulses per trial (three pulses of 1 s duration each) during training, adjusting for the detrimental effect of high shock intensity observed in olfactory relief conditioning [9]. It may be that relatively weak punishment leads to a stronger or quicker relief-effect upon its offset [36]. Our results would support such a mechanism as we could detect the highest relief conditioning score for a longer time delay between shock and color presentation (Fig 2; ISI = +56 s).

Little is known about the neuronal mechanisms underlying olfactory relief conditioning in *Drosophila* [27,37,38]. Fly olfactory relief conditioning seems to differ from both olfactory punishment and reward conditioning with respect to the role of catecholaminergic neurons [37]. However, the role of the *synapsin* gene in the mushroom body neurons appears to be similar between olfactory relief conditioning and punishment conditioning [38].

Interestingly, in rats and humans, relief conditioning engages reward-circuitry [18], although analyses at the level of individual neurons are not feasible. The similarity of the present visual paradigm for relief conditioning in *Drosophila* to rat and human studies may be an advantage in future comparisons of the underlying neural circuits. Regardless, availability of fly relief conditioning paradigms across two different sensory modalities shall be instrumental in revealing conserved mechanisms for memory formation.

Studies across different phyla-i.e., insects, rodents, primates and humans, and across different sensory modalities-i.e., olfactory, visual, [17,18,39–42] reveal that the effect of stimulus timing on memory valence and the learning about sensory traces are shared. The underlying molecular or cellular mechanisms may also be closely related across modalities and animals. Thus, identifying these mechanisms in *Drosophila* may have translational potential.

## Supporting Information

S1 Fig Effect of paired color onset-timing and duration on visual conditioning. Cyan bars indicate the duration of color presentation during training which was either 5 s, 15 s or 25 s-long. Data points indicate the onset of the paired color (ISI = -4 s, -14 s, x-axis) and the mean learning index (y-axis). Error bars represent the SEM. Red stripes indicate electric shock pulses. Learning scores depended on color duration (one-way ANOVA, F = 5.84, p = 0.001), such that prolonged color presentation led to significantly better memory performance (25 s, 15 s *vs*. 5 s) (*post-hoc* pairwise comparisons p < 0.01). All groups showed significant scores (one sample *t*-tests, T > 4.0, p < 0.001), *n* = 20–36.
(TIF)

## References

[1] Rescorla RA. Behavioural studies of Pavlovian conditioning. Ann Rev Neurosci. 1988; 11: 329–352 3284445 10.1146/annurev.ne.11.030188.001553

[2] O’Donnell S, Webb JK, Shine R. Conditioned taste aversion enhances the survival of an endangered predator imperilled by a toxic invader. J Appl Ecol. 2010; 47: 558–565.

[3] Garcia J, Hankins WG, Rusiniak KW. Behavioral regulation of the milieu interne in man and rat. Science. 1974; 185: 824–831. 11785521 10.1126/science.185.4154.824

[4] Gerber B, Yarali A, Diegelmann S, Wotjak CT, Pauli P, Fendt M. Pain-relief learning in flies, rats, and man: basic research and applied perspectives. Learn Mem. 2014; 21: 232–252. 10.1101/lm.032995.113 24643725 PMC3966540

[5] Khurana S, Abu Baker M Bin, Siddiqi O. Odour avoidance learning in the larva of *Drosophila melanogaster* . J Biosci. 2009; 34: 621–631. 19920347 10.1007/s12038-009-0080-9

[6] Tanimoto H, Heisenberg M, Gerber B. Event timing turns punishment to reward. Nature. 2004; 430: 983 15329711 10.1038/430983a

[7] Galili DS, Ludke A, Galizia CG, Szyszka P, Tanimoto H. Olfactory trace conditioning in *Drosophila* . J Neurosci. 2011; 31: 7240–7248. 10.1523/JNEUROSCI.6667-10.2011 21593308 PMC6622595

[8] Shuai Y, Hu Y, Qin H, Campbell R, Zhong Y. Distinct molecular underpinnings of *Drosophila* olfactory trace conditioning. Proc Natl Acad Sci U S A. 2011; 108: 20201–20206. 10.1073/pnas.1107489109 22123966 PMC3250181

[9] Yarali A, Niewalda T, Chen YC, Tanimoto H, Duerrnagel S, et al “Pain relief” learning in fruit flies. Anim Behav. 2008; 76: 1173–1185.

[10] Murakami S, Dan C, Zagaeski B, Maeyama Y, Kunes S, Tabata T. Optimizing *Drosophila* olfactory learning with a semi-automated training device. J Neurosci Methods. 2010; 188: 195–204. 10.1016/j.jneumeth.2010.02.007 20153774 PMC2854214

[11] Solomon RL, Corbit JD. An Opponent-Process theory of motivation: I. Temporal dynamics of affect. Psych Rev. 1974; 81: 119–145. 10.1037/h0036128 4817611

[12] Wagner AR. SOP: a model of automatic memory processing in animal behavior In: Spear NE, Miller RR, editors. Information processing in animals: Memory mechanisms. New Jersey: Erlbaum, Hillsdale 1981; pp. 5–47.

[13] Sutton RS, Barto AG. Time-derivative models of Pavlovian reinforcement Cambridge, MA, US: The MIT Press 1990; pp. 497–537.

[14] Chang RC, Blaisdell AP, Miller RR. Backward conditioning: Mediation by the context. J Exp Psychol Anim Behav Process. 2003; 29: 171–183. 12884677 10.1037/0097-7403.29.3.171

[15] Hellstern F, Malaka R, Hammer M. Backward inhibitory learning in honeybees: a behavioral analysis of reinforcement processing. Learn Mem. 1998; 4: 429–444. 10701882 10.1101/lm.4.5.429

[16] Vogt K, Schnaitmann C, Dylla KV, Knapek S, Aso Y, Rubin GM, et al Shared mushroom body circuits underlie visual and olfactory memories in *Drosophila* . eLife 2014; 3:e02395 10.7554/eLife.02395 25139953 PMC4135349

[17] Andreatta M, Muhlberger A, Yarali A, Gerber B, Pauli P. A rift between implicit and explicit conditioned valence in human pain relief learning. Proc Biol Sci. 2010; 277: 2411–2416. 10.1098/rspb.2010.0103 20356893 PMC2894900

[18] Andreatta M, Fendt M, Mühlberger A, Wieser MJ, Imobersteg S, Yarali A, et al Onset and offset of aversive events establish distinct memories requiring fear and reward networks. Learn Mem. 2012; 19: 518–526. 10.1101/lm.026864.112 23073641

[19] Opfinger E. Ueber die Orientierung der Biene an der Futterquelle. Zeitschrift fuer Vergleichende Physiol. 1931; 15: 431–487.

[20] Menzel R. Das Gedaechtnis der Honigbiene fuer Spektralfarben. Zeitschrift fuer Vergleichende Physiol. 1968; 102: 82–102.

[21] Grossmann KE. Erlernen von Farbreizen an der Futterquelle durch Honigbienen während des Anflugs und während des Saugens. Z Tierpsychol. 1970; 27: 553–562.

[22] Grossmann KE. Belohnungsverzoegerung beim Erlernen einer Farbe an einer kuenstlichen Futterstelle durch Honigbienen. Z Tierpsychol. 1971; 29: 28–41.

[23] Menzel R, Bitterman ME. Learning by honeybees in an unnatural situation Springer-Verlag 1983; pp. 206–215.

[24] Neuser K, Triphan T, Mronz M, Poeck B, Strauss R. Analysis of a spatial orientation memory in *Drosophila* . Nature 2008; 453: 1244–1247. 10.1038/nature07003 18509336

[25] Schnaitmann C, Garbers C, Wachtler T, Tanimoto H. Color discrimination with broadband photoreceptors. Curr Biol. 2013; 23: 2375–2382. 10.1016/j.cub.2013.10.037 24268411

[26] Schnaitmann C, Vogt K, Triphan T, Tanimoto H. Appetitive and aversive visual learning in freely moving *Drosophila* . Front Behav Neurosci. 2010; 4: 10 10.3389/fnbeh.2010.00010 20300462 PMC2839846

[27] Yarali A, Krischke M, Michels B, Saumweber T, Mueller MJ, Gerber B. Genetic distortion of the balance between punishment and relief learning in *Drosophila* . J Neurogenet. 2009; 23: 235–247. 10.1080/01677060802441372 19052955

[28] Yarali A, Nehrkorn J, Tanimoto H, Herz AVM. Event timing in associative learning: From biochemical reaction dynamics to behavioural observations PLoS One 2012; 7(3): e32885 10.1371/journal.pone.0032885 22493657 PMC3316544

[29] Burman MA, Gewirtz JC. Timing of fear expression in trace and delay conditioning measured by fear-potentiated startle in rats. Learn Mem. 2004; 11: 205–212. 15054136 10.1101/lm.66004 PMC379691

[30] Pavlov IP. Conditioned Reflexes: An investigation of the physiological activity of the cerebral cortex Oxford University Press 1927. 10.5214/ans.0972-7531.1017309 PMC4116985 25205891

[31] Chittka L, Niven J. Are bigger brains better? Curr Biol. 2009; 19: R995–R1008. 10.1016/j.cub.2009.08.023 19922859

[32] Aso Y, Siwanowicz I, Bräcker L, Ito K, Kitamoto T, Tanimoto H. Specific dopaminergic neurons for the formation of labile aversive memory. Curr Biol. 2010; 20: 1445–1451. 10.1016/j.cub.2010.06.048 20637624 PMC2929706

[33] Claridge-Chang A, Roorda RD, Vrontou E, Sjulson L, Li H, Hirsh J, et al Writing memories with light-addressable reinforcement circuitry. Cell 2009; 139: 405–415. 10.1016/j.cell.2009.08.034 19837039 PMC3920284

[34] Aso Y, Sitaraman D, Ichinose T, Kaun KR, Vogt K, Belliart-Guérin G, et al Mushroom body output neurons encode valence and guide memory-based action selection in Drosophila. eLife 2014; 3:e04580 10.7554/eLife.04580 25535794 PMC4273436

[35] Dylla K V, Galili DS, Szyszka P, Lüdke A. Trace conditioning in insects-keep the trace! Front Physiol. 2013; 4: 67 10.3389/fphys.2013.00067 23986710 PMC3750952

[36] Franklin JC, Lee KM, Hanna EK, Prinstein MJ. Feeling worse to feel better: pain-offset relief simultaneously stimulates positive affect and reduces negative affect. Psychol Sci. 2013; 24: 521–529. 10.1177/0956797612458805 23459871

[37] Yarali A, Gerber B. A neurogenetic dissociation between punishment-, reward-, and relief-learning in *Drosophila* . Front Behav Neurosci. 2010; 4: 189 10.3389/fnbeh.2010.00189 21206762 PMC3013555

[38] Niewalda T, Michels B, Jungnickel R, Diegelmann S, Kleber J, Kähne T, et al Synapsin determines memory strength after punishment- and relief-learning. J Neurosci. 2015; 35 (19): 7487–7502. 10.1523/JNEUROSCI.4454-14.2015 25972175 PMC4429153

[39] Bitterman ME, Menzel R, Fietz A, Schäfer S. Classical conditioning of proboscis extension in honeybees (*Apis mellifera*). J Comp Psychol. 1983; 97: 107–119. 6872507

[40] Rogan MT, Leon KS, Perez DL, Kandel ER. Distinct neural signatures for safety and danger in the amygdala and striatum of the mouse. Neuron. 2005; 46: 309–320. 15848808 10.1016/j.neuron.2005.02.017

[41] Belova MA, Paton JJ, Morrison SSE, Salzman CD. Expectation modulates neural responses to pleasant and aversive stimuli in primate amygdala. Neuron. 2007; 55: 970–984. 17880899 10.1016/j.neuron.2007.08.004 PMC2042139

[42] Seymour B, O’Doherty JP, Koltzenburg M, Wiech K, Frackowiak R, Friston K, et al Opponent appetitive-aversive neural processes underlie predictive learning of pain relief. Nat Neurosci. 2005; 8: 1234–1240. 16116445 10.1038/nn1527

