# Supplementary figures and images for: Reversing Stimulus Timing in Visual Conditioning Leads to Memories with Opposite Valence in Drosophila
Source: PLoS One. 2015 Oct 2;10(10):e0139797. doi: 10.1371/journal.pone.0139797 (PMC4592196; doi:10.1371/journal.pone.0139797)

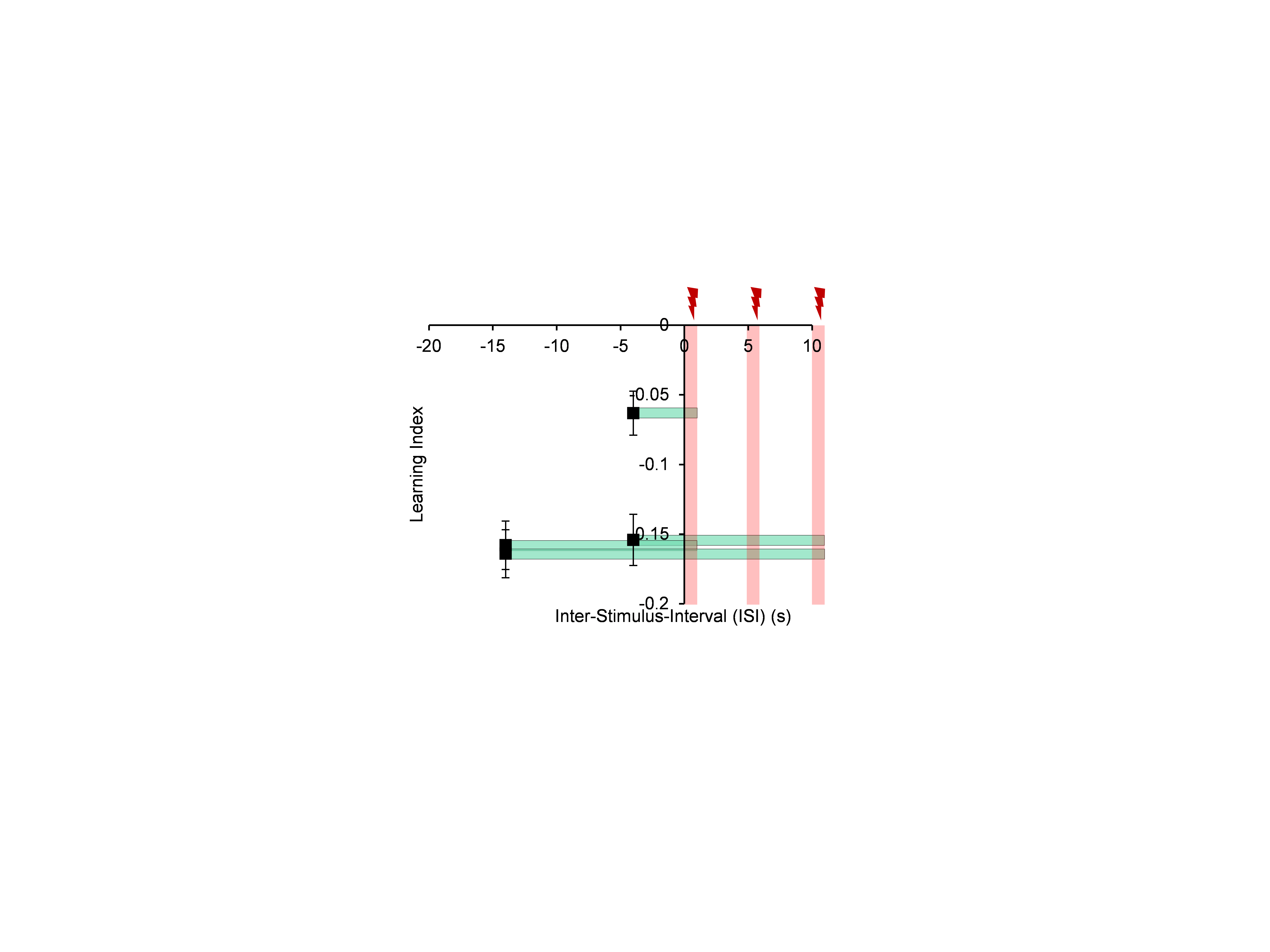

Supplement: S1 Fig — Cyan bars indicate the duration of color presentation during training which was either 5 s, 15 s or 25 s-long. Data points indicate the onset of the paired color (ISI = -4 s, -14 s, x-axis) and the mean learning index (y-axis). Error bars represent the SEM. Red stripes indicate electric shock pulses. Learning scores depended on color duration (one-way ANOVA, F = 5.84, p = 0.001), such that prolonged color presentation led to significantly better memory performance (25 s, 15 s vs. 5 s) (post-hoc pairwise comparisons p < 0.01). All groups showed significant scores (one sample t-tests, T > 4.0, p < 0.001), n = 20–36. (TIF) [file pone.0139797.s001.tif]
